# Cytological basis of sterility in male and female hybrids between sibling species of grey voles *Microtus arvalis* and *M. levis*

**DOI:** 10.1038/srep36564

**Published:** 2016-11-04

**Authors:** Anna A. Torgasheva, Pavel M. Borodin

**Affiliations:** 1 Institute of Cytology and Genetics, Russian Academy of Sciences, Siberian Department, Novosibirsk 630090, Russia; 2 Novosibirsk State University, Novosibirsk, Russia

## Abstract

To make insight into the cytological basis of reproductive isolation, we examined chromosome synapsis and recombination in sterile male and female hybrids between *Microtus arvalis* and *M. levis.* These sibling species differ by a series of chromosomal rearrangements (fusions, inversions, centromere shifts and heterochromatin insertions). We found that meiosis in male hybrids was arrested at leptotene with complete failure of chromosome pairing and DNA double-strand breaks repair. In the female hybrids meiosis proceeded to pachytene; however, the oocytes varied in the degree of pairing errors. Some of them demonstrated almost correct chromosome pairing, while most of them contained a varying number of univalents and multivalents with extensive regions of asynapsis and non-homologous synapsis. Variation between oocytes was probably caused by stochasticity in the ratio of homologous to non-homologous pairing initiations. We suggest that substantial chromosomal and genetic divergence between the parental species affects preliminary alignment of homologues, homology search and elimination of ectopic interhomologue interactions that are required for correct homologous pairing. Apparently, pairing failure in male and aberrant synapsis in female vole hybrids followed by meiotic silencing of unsynapsed chromatin cause apoptosis of gametocytes and sterility.

Homologous chromosome recombination in meiotic prophase is required for orderly chromosome segregation. Recombination is preceded by chromosome prealignment and the scheduled formation of DNA double-strand breaks (DSBs), followed by a RAD51-mediated search for homologous DNA sequences and the formation of heteroduplexes involving DNA strands of homologous chromosomes at early stages of meiotic prophase (leptotene and zygotene). Polymerisation of the synaptonemal complex (SC), a meiotic-specific proteinaceous structure, stabilises these connections and completes homologous chromosome synapsis. A small proportion of DSBs (at least one per chromosome pair) is repaired as crossovers (reciprocal exchanges between homologues). The sites of crossing over can be visualised in mid-meiotic prophase (pachytene) as late recombination nodules containing MLH1 (mismatch repair protein), and at diplotene-diakinesis as chiasmata. Sister chromatid cohesion beyond the chiasmata holds homologues together at metaphase-I, ensuring proper orientation and orderly segregation^1^,^2^.

These complex and highly coordinated processes are thoroughly checked by natural selection at each meiosis, and so the genetic unity of the species is preserved. The evolution of geographically isolated populations, however, leads to the fixation of novel chromosomal rearrangements and a divergence of the factors controlling DSB formation and the DNA sequences involved in homology search. In hybrids, karyotypic and genetic divergence can result in meiotic aberrations and variable degrees of infertility due to germ-cell death or the formation of unbalanced gametes.

Although mammalian hybrids have been known for centuries^3^, studies of the genetic and cellular bases of hybrid sterility in mammals are surprisingly scarce. Several genes causing male sterility in hybrids between karyotypically identical species of the house mouse (*Mus musculus x M. domesticus*)^5^,^6^,^7^,^8^ and felines (*Felis catus x Profelis serval, F. catus x Prionailurus bengalensis*)^9^ have been localised and sequenced. One of them, PRDM9 (referred to as a speciation gene), is involved in the control of DSB formation^10^. Male sterility or reduced fertility accompanied by synaptic aberrations of autosomes and sex chromosomes has been widely reported in hybrids of chromosomally divergent mammalian species, subspecies and local populations^3^,^11^,^12^,^13^,^14^,^15^,^16^,^17^,^18^,^19^.

Sterility in mammalian hybrids is in a good agreement with Haldane’s rule that “when in the F1 offspring of a cross between two animal species or races one sex is absent, rare, or sterile, that sex is always the heterozygous sex”^20^. Several hypotheses have been proposed to explain the genetic basis of this rule. Among them, the dominance and “faster male” hypotheses are considered the most plausible^21^,^22^,^23^,^24^,^25^. The dominance model ascribes the predominant inviability/sterility of the heterogametic sex to the alternative fixation of X-linked recessive mutations. The higher the ratio between recessive and dominant mutations of incompatibility, the larger the time lag between hetero- and homogametic sexes in the evolution of hybrid unfitness^26^. The “faster male” hypothesis suggests that male fertility alleles evolve and diverge faster due to divergent sexual selection. At the same time, strong sperm selection leads to increase of the stringency of male meiotic checkpoints^25^,^27^. In mammals, male meiosis is very sensitive to genetic and chromosomal aberrations. Meiotic mutants and knockouts affect meiosis earlier and more severely in males than in females^28^ and this amplifies the effects of genetic and chromosomal incompatibility on the fertility of male hybrids.

Haldane’s rule usually applies only to early stages of speciation. Building reproductive barriers is a snowball process^29^, or, as Darwin (1866) put it, a series of “graduated steps from very slightly lessened fertility to utter and absolute sterility”^30^. Therefore, at advanced stages of reproductive isolation, both sexes are sterile. We are currently unclear, however, as to whether males and females proceed to complete sterility by the same or by different routes. Sex differences in meiotic disruption within hybrids, then, can tell us about the genetic and cytological bases of these steps. Unfortunately, due to technical difficulties female meiosis in the hybrids has rarely been analysed. We are aware of only one such study^31^, which found that female hybrids between *Mus musculus* and *M. domesticus* were fertile, even though their oocytes displayed the same pairing abnormalities as male hybrids - with half the frequency, though.

To make insight into the cytological basis of hybrid sterility, we examined chromosome synapsis and recombination in male and female sterile hybrids between two sibling species of the grey vole, *Microtus arvalis* (dams) and *M. levis* (sires). These species diverged from 0.5 to 4.3 MYA^32^,^33^, differ by a series of chromosomal rearrangements^33^,^34^, yet remain morphologically indistinguishable^35^. In nature, hybrids occur at the zone of sympatry in the Urals, and can easily be produced in laboratory settings^35^,^36^,^37^,^38^,^39^. Reciprocal hybrids between *M. arvalis* and *M. levis* are completely sterile; however, males differ from females in the stage of reproductive collapse^38^. In males, testis mass is severely reduced and no sperm is found in the epididymis. Male meiosis arrests at prophase I but occasional nuclei reach diakinesis-metaphase I, where they mainly show univalents. More advanced stages of spermatogenesis have not been detected^40^. In female hybrids, oocyte growth and development was described as normal. However, follicular atresia was detected in hybrids even at the primordial follicle stage, and, at all stages, was more pronounced than in females of the parental species. Hybrid females display abnormal ovulation without follicle wall breakage. Mature oocytes move into Graafian follicles where they undergo the second meiotic division. No *corpus luteum* was detected in hybrid females^41^. According to Gileva *et al*.^37^ backcross progeny may be produced very rarely, but in our hybridisation experiments none occurred. Thus, these hybrids provide a promising model for studying cellular mechanisms of male and female hybrid sterility.

We analysed key events of chromosome synapsis and recombination. DSBs were detected by immunolocalisation of RAD51, a marker for single-stranded DNA ends^42^, and γH2A.X, a phosphorylated form of histone H2A.X^43^. Polymerisation of the lateral elements of SC and the formation of its central element were visualised with antibodies to SYCP3 and SYCP1^43^. The number and distribution of recombination events were estimated by immunolocalisation of MLH1^44^. This analysis allowed us to detect meiotic aberrations leading to hybrid sterility.

## Results

### Parental species

**Figure 1:**
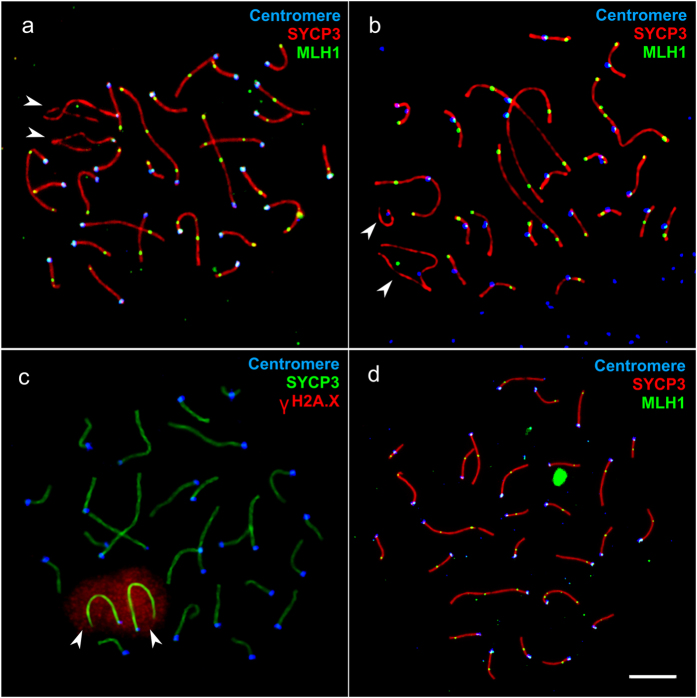
Pachytene spermatocytes of *M. levis* (**a,c**) and *M. аrvalis* (**b**) and an oocyte of *M. levis* (**d**). Arrowheads indicate unpaired sex chromosomes. Bar – 5 μm.

Chromosome pairing and recombination in male *M. arvalis* and *M. levis* have been described previously^45^,^46^,^47^. Both species showed asynapsis of the X and Y chromosomes (Fig. 1a,b, Supplementary Fig. S1) which underwent meiotic sex chromosome inactivation and were labeled by γH2A.X antibodies (Fig. 1c, Fig. S1). This feature is characteristic of the entire *arvalis* lineage of the genus Microtus^47^. Asynapsis of autosomes at pachytene stage, when most chromosomes contained MLH1 foci, was very rare (less than 2% in both sexes) and always affected small chromosomes. *M. arvalis* and *M. levis* males did not differ from each other in total autosomal SC length (t_212,1_ = 2.6, P = 0.09) or MLH1 focus number per cell (t_212,1_ = 1.1, P = 0.29) (Table 1). The number of MLH1 foci was usually restricted to one per autosomal bivalent in spermatocytes, as was in oocytes (Fig. 1d, Table 1). Assuming at least one focus on the XX bivalent, no significant sex differences in recombination rates can therefore be seen in *M. levis.*

By contrast, a drastic sex difference in chromosome behavior is observed in hybrids, in which oocytes show almost normal progression until late pachytene while most spermatocytes are arrested at leptotene.

### Female hybrids

**Figure 2:**
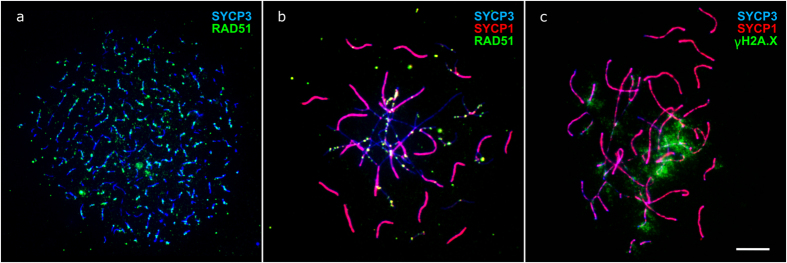
Leptotene (**a**) and zygotene (**b,c**) oocytes of F1 hybrids between *M. аrvalis* and *M. levis*. Bar – 5 μm. (**a**) Assembling lateral elements of the SC (revealed by SYCP3 antibodies) accompanied by extensive RAD51 labeling. (**b,c**) Asynchronous formation of central elements of the SC. At completely pared SC blue signal of SYCP3 is co-localised with red signal of SYCP1. Regions with delayed synapsis are marked with RAD51 and γH2A.X antibodies.

Leptotene oocytes of hybrids appeared normal with assembling lateral elements of SC labeled by RAD51 (Fig. 2a). At zygotene, lateral elements established contacts with each other while the central elements formed asynchronously. Thus, while some lateral elements were already paired, others displayed extensive asynapsis. Asynaptic regions were often intertwined and labeled by RAD51 and γH2A.X (Fig. 2b,c, Fig. S2). Some regions kept these marks until pachytene when most autosomes were synapsed (Fig. S2).

**Figure 3:**
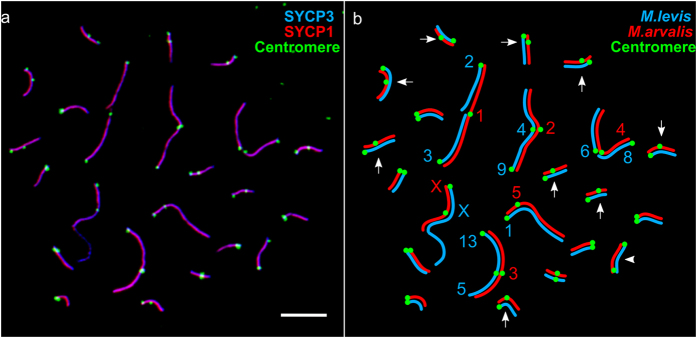
A microphotograph (**a**) and a schematic (**b**) of a completely paired SC complement in a pachytene oocyte of an F1 hybrid between *M. arvalis* and *M. levis*. Bar – 5 μm. (**a**) Red and blue layers are slightly shifted in the merged image to show complete co-localisation of SYCP1 (central element) and SYCP3 (lateral elements of SC) in all synaptic configurations, except the heterochromatic block of the *M. levis* X chromosome. (**b**) Diagram of *M. arvalis-* (red) and *M. levis-* (blue) derived lateral elements, suggested by comparative metaphase analysis of the parental karyotypes. Arrows show bivalents with centromere signals shifted from each other, and an arrowhead shows a bivalent with centromere signals at both ends.

Pachytene oocytes were very variable in their appearance, containing asynaptic and heterosynaptic configurations. However, about 10% of oocytes had 23 “correctly” paired configurations, with four large trivalents, twelve heteromorphic and seven homomorphic bivalents (six acrocentric and one metacentric) (Fig. 3a). The heteromorphic bivalents comprised the X pair with long arms of different length and misaligned centromeres, and one large and ten smaller bivalents with the two centromere signals shifted. This is in a good agreement with pairing expected from comparative metaphase analysis of the parents (Fig. 3b). Parental karyotypes differ by at least four tandem fusions (producing trivalents in F1 hybrids), one paracentric and six pericentric inversions (which may produce inversion loops or straight bivalents with misaligned centromeres), four to seven putative centric transpositions (producing bivalents with misaligned centromeres), and an insertion of a large heterochromatic block in the X of *M. levis*^33^,^34^.

Although good correspondence between expected and observed synaptic configurations was found, there were two discordances. One heteromorphic bivalent showed centromere signals on its both ends (Fig. 3b, arrowhead). A trivalent comprising metacentric chromosome 1 of *M. arvalis* and twin acrocentrics of *M. levis* contained three misaligned centromeres. Lemskaya *et al*.^34^ proposed that chromosome 1 of *M. arvalis* resulted from tandem fusion of the proximal end of chromosome 3 and the distal end of chromosome 2 of *M. levis*. However, the synaptic configurations observed in these F1 hybrids indicate that chromosome 1 of *M. arvalis* was generated by fusion of the distal ends of the acrocentrics followed by inactivation of their centromeres, and re-activation of the centromere at the point of fusion.

**Figure 4:**
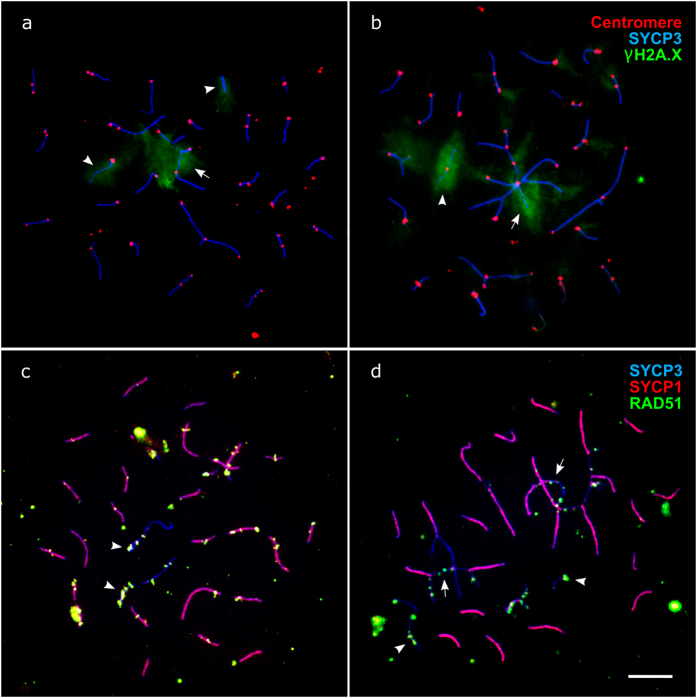
Synaptic aberrations in pachytene oocytes of F1 hybrids between *M. аrvalis* and *M. levis*. Univalents (arrowheads) and unpaired regions in multivalents (arrows) contain unrepaired DSBs, revealed by antibodies to γH2A.X (**a,b**) and RAD51 (**c,d**). Bar – 5 μm.

**Figure 5:**
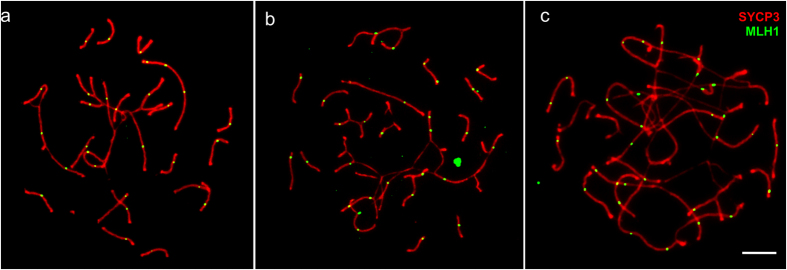
Immunolocalisation of MLH1 and SCs in pachytene oocytes of F1 hybrids between *M. аrvalis* and *M. levis,* containing one complex multivalent (**a**), several multivalents (**b**), and long asynaptic regions (**c**). Bar – 5 μm.

More than 90% of pachytene cells in female hybrids contained synaptic aberrations. Supplementary Fig. S2 shows examples of these aberrations. The most common were multivalents containing more than three lateral elements (from four to twenty, 6.5 ± 3.4 on average) (Figs 4 and 5). The number of multivalents varied from one to four per cell (1.7 ± 0.9 on average) and most multivalents were composed of large chromosomes. The probability for large metacentric chromosomes 1 to 4 of *M. arvalis* and the acrocentric pairs of *M. levis* to be involved in multivalent formation was 0.60 ± 0.06. This probability was also high for the X chromosomes (0.42 ± 0.11) and submetacentric chromosome 5 of *M. arvalis* with homologous acrocentric chromosome 1 of *M. levis* (0.32 ± 0.11). The probability for medium sized and small chromosomes, however, was very low (0.05 ± 0.01).

Univalents were also seen, varying in number from one to nine (3.0 ± 2.4 on average). In many cells odd numbers of univalents were found, indicating that some of their partners were involved in non-homologous synapsis in a multivalent. For the same reason the average number of observed trivalents was lower than the expected four (2.5 ± 1.4 on average). The number of bivalents in oocytes was lower than the expected 19 (15.4 ± 1.5 on average), again due to involvement of one or both partners in non-homologous synapsis in multivalents. Asynapsis was frequently observed around pairing partner switch points and at the ends of multivalent chains. Unpaired regions were labeled with γH2A.X (Fig. 4a,b, Fig. S2) and RAD51 (Fig. 4c,d, Fig. S2), indicating that they contained unrepaired DSBs.

Figure 5 and Fig. S2 show immunolocalisation of MLH1 at the SCs in pachytene oocytes of F1 hybrids between *M. аrvalis* and *M. levis*. The mean number of MLH1 foci was significantly lower in hybrid oocytes than in oocytes of *M. levis* (t_96,1_ = 9.6, P < 0.001: Table 1), while there was no difference in total SC length (t_78,1_ = 1.0, P = 0.32: Table 1). The decrease in hybrids was mainly due to a lack of foci in univalents. There was no substantial decrease in recombination between the homologous segments in homomorphic and heteromorphic bivalents, trivalents or multivalents. Most paired arms had at least one MLH1 focus. Even in the cells containing very complex multivalents (Fig. 5a), a series of multivalents (Fig. 5b), or long asynaptic regions (Fig. 5c) we did not observed a substantial decrease in the number of MLH1 foci.

### Male hybrids

**Figure 6:**
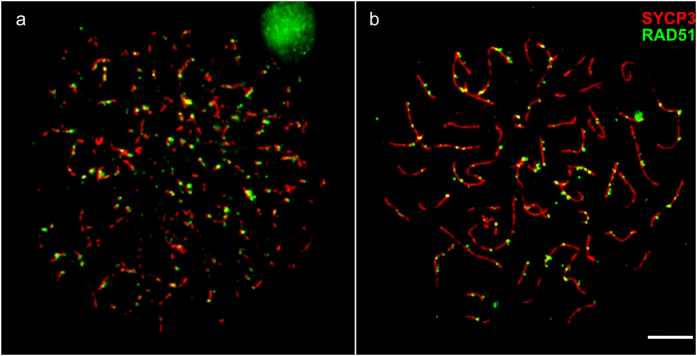
Leptotene spermatocytes of F1 hybrids between *M. аrvalis* and *M. levis*. Bar – 5 μm. (**a**) A typical spermatocyte with incompletely assembled axial elements. (**b**) One of the most advanced spermatocytes with almost completely assembled axial elements.

Male meiosis in hybrids was generally arrested at leptotene. The majority of spermatocytes carried only fragments of the axial elements (Fig. 6a, Fig. S2), indicating incomplete assembly. The most advanced spermatocytes contained almost normal axial elements, but even in these cells no initiation of synapsis was seen (Fig. 6b). The halved total length of the lateral elements in the male hybrids at this stage was about 10% longer than the total SC length of the male *M. levis* at pachytene (t_92,1_ = 4.4, P < 0.001: Table 1). Hybrid spermatocytes showed heavy labeling with RAD51 antibodies indicating DSB presence.

## Discussion

This study supports breeding records and histological assessments showing the sterility of hybrids between *M. arvalis* and *M. levis*^35^,^36^,^37^,^38^,^39^,^41^. Hybrids of both sexes are sterile; however, males, in accordance with Haldane’s rule, are “more sterile” than females. Male meiosis is uniformly arrested at leptotene, while female meiosis is affected at pachytene with wide variation between oocytes in the degree of disturbances of chromosome pairing. This might be due to the sex difference in degree of methylation on the meiotic onset. Studies in mice demonstrated that female germ cells enter meiosis in a demethylated state, while the genome of male germ cells is heavily remethylated after mitotic arrest at the beginning of meiosis^48^,^49^,^50^. A faster evolution of male hybrid sterility in the grey voles might also be accelerated by constitutive asynapsis and total lack of recombination between XY in this phylogenetic lineage^47^.

Several features of meiotic disturbances in female hybrids between *M. arvalis* and *M. levis* also characterise male meiosis in many cases of male-only hybrid sterility. The zygotene-pachytene transition is mainly affected, asynapsis and heterosynapsis occur in both heteromorphic and homomorphic chromosomes, while cells vary widely in the degree of disturbances and the number of chromosomes affected^11^,^12^,^51^,^52^,^53^.

Despite the sex differences in the stage and degree of meiotic disturbances, the cause of these disturbances is probably the same. This is failure of a homologous synapsis between homologous chromosome regions, which is complete in males and partial and sporadic in females. The majority of oocytes contains multiple regions of asynapsis and non-homologous synapsis. The establishment and extension of non-homologous synapsis leads to multivalent and univalent formation. Thus, when one homologue is involved in a multivalent, the other either remains univalent or pairs non-homologously with another chromosome region in the same or a different multivalent.

Homologous pairing involves several steps: preliminary DSB-independent pairing, DSB-mediated homology search with formation of interhomologue interactions, and elimination of unwanted contacts between nonhomologous chromosomes^1^.

DSB-independent pairing probably depends on associations of centromeres and/or telomeres at early prophase. It restricts the searching area for homologous recognition and alignment^2^. This process may be impaired in grey vole hybrids, which are heterozygous for at least 15 chromosomal rearrangements^33^,^34^. Although heterozygosity for a single fusion, pericentric inversion or centromeric shift does not usually lead to pairing abnormalities^45^,^54^,^55^,^56^, a high number of such heterozygosities may impede or delay presynaptic alignment of homologous regions^57^. It has been shown that multiple heterozygosity for a series of Robertsonian translocations in mice alters the nuclear architecture characteristic of the telocentric karyotype, changes patterns of centromere clustering and other interactions between chromosomal domains and leads to ectopic associations^58^. Similar problems in centromere clustering should occur in multiple heterozygotes for pericentric inversions and centromeric shifts.

Insufficient pre-DSB coalignment in the hybrids may enhance the probability of entanglements between chromosomes and increase the occurrence of ectopic recombinational intermediates between nearly homologous sites of nonhomologous chromosomes. Because the sequence homology matching in these intermediates is apparently low, they should be less stable. The unwanted DNA connections tend to be eliminated by the mismatch repair system^59^ and active chromosome movements^62^. We propose that the balance in stability between “wanted” and “unwanted” connections is impaired in hybrids, due to a decrease of sequence homology between the homologous regions of the parental species. This leads to variability between oocytes in the number and size of regions involved in multivalents.

These requirements of normal synapsis between homologous chromosomes are apparently not met in the hybrids. Multiple heterozygosity for chromosomal rearrangements hinders preliminary DSB-independent pairing between homologous chromosomes and increases the incidence of non-homologous associations. Divergence between parental genomes decreases homology at the sequence level, the stability of homologous heteroduplexes, and affects the efficiency of discrimination between correct and ectopic interhomologue interactions. In hybrids, the wide variation between genetically and chromosomally identical oocytes in the ratio of homologously paired to non-homologously paired regions indicates that the choice between homologous and non-homologous synapsis at each of these steps is random. Detailed molecular mechanisms of these processes remain to be elucidated, and interspecies hybrids, such as those reported here, provide an excellent model for future studies.

The sex difference observed in this study can be categorised as an example of “graduated steps of sterility”^30^ from advanced in females to complete in males. Genetic and chromosomal incompatibility is probably amplified in the male hybrids by the well known vulnerability of spermatogenesis to pairing aberrations^28^. We observed stochastic variation in “degree of sterility” even between oocytes of the same F1 genotype. Those containing “correctly” paired configurations are probably able to produce viable balanced oocytes. The more asynapsed regions an oocyte contain, the larger part of its genome undergoes meiotic silencing of unsynapsed chromatin (MSUC)^64^,^65^, and the higher the chance for the oocyte to be directed to apoptosis. The results of this study indicate that reproductive isolation based on hybrid sterility may be built up in a gradual mode. A gradual genetic divergence and the sequential fixation of different chromosome rearrangements in isolated populations increase the probability of pairing errors followed by MSUC and apoptosis in the hybrid gametocytes.

## Materials and Methods

Seven adult male and 12 newborn female hybrids between *M. arvalis* (dams) and *M. levis* (sires) were examined, as well as three adult male *M. arvalis,* four adult male and three newborn female *M. levis*. Captive-bred colonies of the parental species were established from individuals trapped in Leningrad district (*M. arvalis*) and Novosibirsk district (*M. levis*) and maintained in the animal house of the Institute of Cytology and Genetics. Maintenance, handling and euthanasia of animals followed protocols approved by the Animal Care and Use Committee of the Institute of Cytology and Genetics. Experiments described in this manuscript were carried out in accordance with the approved national guidelines for the care and use of laboratory animals.

Chromosome spreads were prepared from spermatocytes or embryonic oocytes according to Peters *et al*.^66^. Cell spreads were treated as described in Anderson *et al*.^44^ using rabbit polyclonal anti-SYCP3 (1:500; Abcam), mouse monoclonal anti-SYCP3 (1:100; Abcam), rabbit polyclonal anti-SYCP1 (1:500; Abcam), mouse monoclonal anti-MLH1 (1:50; Abcam), rabbit polyclonal anti-RAD51 (1:200; Calbiochem), mouse monoclonal anti-γH2A.X (1:500; Abcam), rabbit polyclonal anti-γH2A.X (1:500; Abcam) and human anticentromere (ACA) (1:100; Antibodies Inc) primary antibodies. The secondary antibodies used were Cy3-conjugated goat anti-rabbit (1:500; Jackson ImmunoResearch), Alexa450-conjugated goat anti-rabbit (1:100; Invitrogen), FITC-conjugated donkey anti-rabbit (1:200; Jackson ImmunoResearch), FITC-conjugated goat anti-mouse (1:50; Jackson ImmunoResearch), AMCA-conjugated donkey anti-human (1:100; Jackson ImmunoResearch), and Cy3-conjugated goat anti-human (1:100; Jackson ImmunoResearch) antibodies. Antibodies were diluted in PBT (3% bovine serum albumin and 0.05% Tween 20 in phosphate-buffered saline). A solution of 10% PBT was used for blocking. Primary antibody incubations were performed overnight in a humid chamber at 37 °C; and secondary antibody incubations, for 1 h at 37 °C. Slides were mounted in Vectashield antifade mounting medium (Vector Laboratories) to reduce fluorescence fading.

Preparations were visualised with an Axioplan 2 microscope (Carl Zeiss) equipped with a CCD camera (CV M300, JAI), CHROMA filter sets and an ISIS4 image processing package (MetaSystems GmbH).

Centromeres were identified by ACA foci. MLH1 signals were scored only if they were localised on the SC. In the parental species the length of the SC of all bivalents was measured in micrometers using MicroMeasure 3.3^67^. To estimate the total SC length in the hybrids, whose pachytene cells contained partially or completely unpaired chromosomes, we measured the lateral elements of SC and then divided the sum by two.

Statistica 6.0 software package (StatSoft, Tulsa, OK, USA) was used for descriptive statistics. For the comparisons of SC length and MLH1 foci number between parental species and their F1 hybrids, Students’ *t*-tests (two-sided) were performed.

## Additional Information

**How to cite this article**: Torgasheva, A. A. and Borodin, P. M. Cytological basis of sterility in male and female hybrids between sibling species of grey voles *Microtus arvalis* and *M. levis*. *Sci. Rep.* **6**, 36564; doi: 10.1038/srep36564 (2016).

**Publisher’s note:** Springer Nature remains neutral with regard to jurisdictional claims in published maps and institutional affiliations.

## Supplementary Material

Supplementary Information

## Figures and Tables

**Table 1:** The total length of SC and the number of MLH1 foci (mean  ±  S.D.) per cell in *M. arvalis, M. levis* and their F1 hybrids.

Genotype	n	SC length, μm	n	MLH1 foci number
*M. arvalis,* males	150	144.7 ± 14.8	150	27.6 ± 1.2
*M. levis,* males	64	150.9 ± 17.6	64	27.8 ± 1.4
*M. levis,* females	50	163.6 ± 21.2	58	28.8 ± 1.4
F1 hybrids, males	30	170.4 ± 24.2	50	0
F1 hybrids, females	30	168.7 ± 23.5	40	24.6 ± 2.9

n - number of cells examined.
