# Supplementary material for: Cytological basis of sterility in male and female hybrids between sibling species of grey voles Microtus arvalis and M. levis
Source: Sci Rep. 2016 Nov 4;6:36564. doi: 10.1038/srep36564 (PMC5109913; doi:10.1038/srep36564)
Supplement: Supplementary Information [file srep36564-s1.pdf]

Supplementary Information

Cytological basis of sterility in male and female hybrids between sibling species of grey voles *Microtus arvalis* and *M. levis*

Anna A. Torgasheva[1,2], Pavel M. Borodin[1*,2]

[1] *Institute of Cytology and Genetics, Russian Academy of Sciences, Siberian Department, Novosibirsk 630090, Russia*

[2] *Novosibirsk State University, Novosibirsk, Russia*

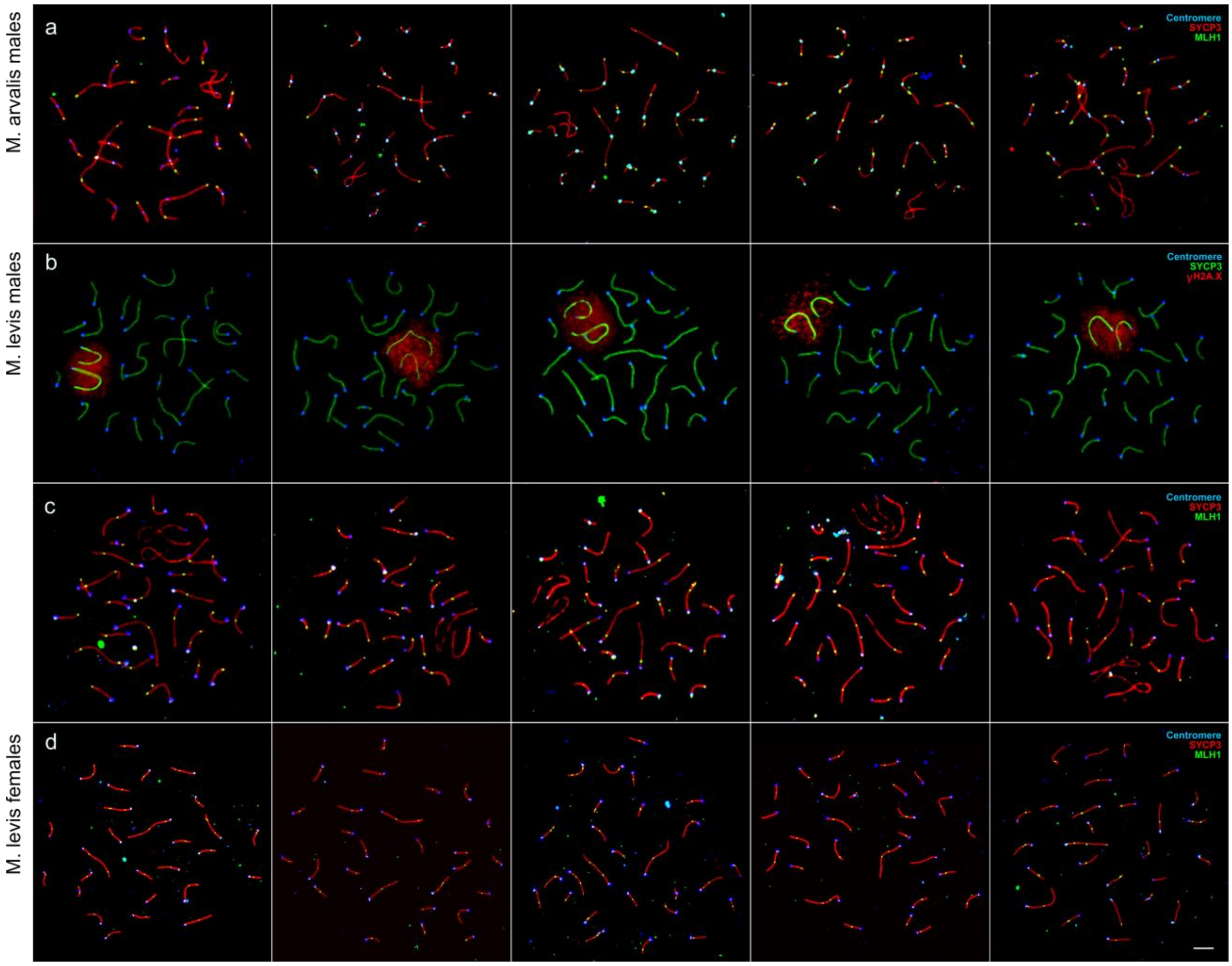

Figure S1. Pachytene spermatocytes of *M. arvalis* (a) and *M. levis* (b, c) and oocytes of *M. levis* (d). Bar – 5 µm.

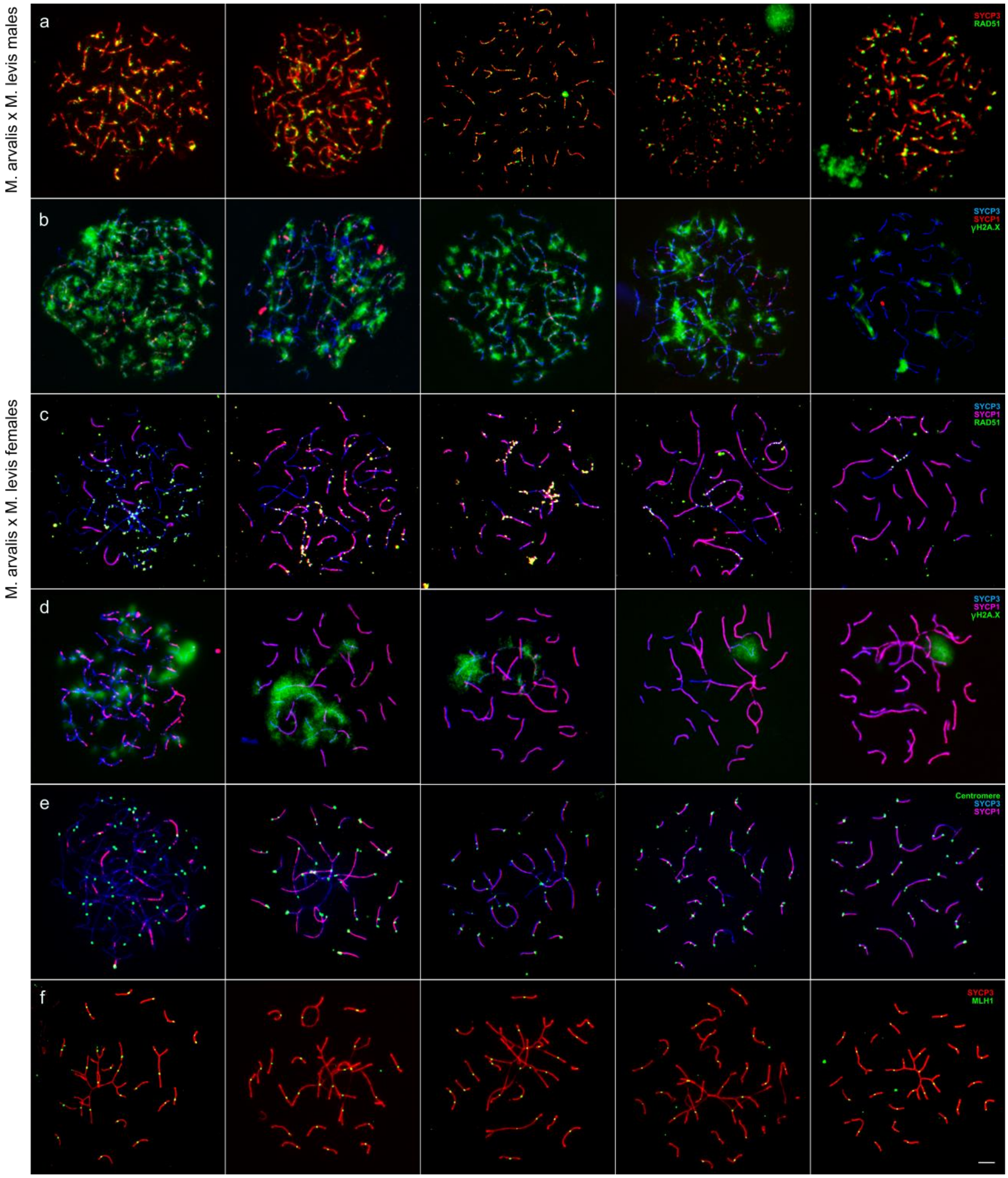

Figure S2. Variation of synaptic configurations in male (a, b) and female (c-f) F1 hybrids between *M. arvalis* and *M. levis*. Bar – 5 μm.
